# Influenza vaccination among health care workers in Thailand

**DOI:** 10.1186/1753-6561-5-S6-P84

**Published:** 2011-06-29

**Authors:** T Chotpitayasunondh, N Sawanpanyalert, R Bumrungsak, P Chunthitiwong, P Chainatraporn

**Affiliations:** 1 Medical Services, Queen Sirkit National Insitute of Child Health, Bangkok, Thailand

## Introduction / objectives

To determine the knowledge, attitudes, and practice related to influenza vaccinations of health care workers (HCWs).

## Methods

Between May 2008 and December 2009, a self-administered questionnaire was distributed to HCWs participating in one of the 5 two-day training courses for emerging infectious diseases in several regional health jurisdictions of Thailand.

## Results

Of 1466 participants, the response rate of questionnaire was 53.8%. The respondents were physicians (14.8%), nurses (60.2%), and other groups of healthcare professionals (25%). The vaccination rate among nurses (92.8%) was statistically significantly higher than among physicians (86.2%) and other groups (81.1%) (*p*<0.001). The overall influenza vaccination rate was 89%. The awareness of both the health benefit of this vaccine (57.2%) and their heightened risk of acquiring influenza (41.1%) were the two most common reasons for vaccine acceptance. The most common reason for vaccine refusal was the belief that, without underlying disease or co-morbidity, the vaccine was unnecessary (43.2%). Further, the fear of potentially serious adverse effects of the vaccine was also reported as a common reason for not being vaccinated (31.8%). The vaccinated group was significantly more likely to encourage their family members to receive this vaccine (74.6% ) as compared to the non-vaccinated group ( 45.6%) (*p* <0.001).

## Conclusion

We identified a relatively high coverage of influenza vaccination among HCWs in Thailand. The complacency about their potential risk of influenza complication and the fear of vaccine adverse effect were the major barriers to influenza vaccination. Thus, the provision of proper education program on influenza vaccination is essential for the successful immmunization campaign for HCWs.

## Disclosure of interest

T. Chotpitayasunondh Employee of Government.

